# Supplementary material for: Potentially toxic metal(loid) distribution and migration in the bottom weathering profile of indigenous zinc smelting slag pile in clastic rock region
Source: PeerJ. 2021 Apr 7;9:e10825. doi: 10.7717/peerj.10825 (PMC8035896; doi:10.7717/peerj.10825)
Supplement: Supplemental Information 1 [file peerj-09-10825-s001.doc]

Table S1 Soil characteristics and potentially toxic metal(loid) concentrations in both slag-covered and slag-absent weathering profile in the study area

| Units | | | | value | mg/kg | mg/kg | mg/kg | mg/kg | mg/kg | mg/kg | mg/kg | % |
| --- | --- | --- | --- | --- | --- | --- | --- | --- | --- | --- | --- | --- |
| Lowest limits of quantifications | | | | 0.01 | 0.2 | 0.02 | 1 | 0.2 | 0.004 | 0.5 | 2 | 0.01 |
| Soil profile | Characteristics of soil profile | Sample number and sampling depth (cm) | Characteristics of sample | pH | As | Cd | Cr | Cu | Hg | Pb | Zn | Al |
| Slag-covered | In clastic rock region, covered by the indigenous zinc smelting zinc slag | Slag | Dark gray silt with fragments of slag | 6.83 | 636.0 | 192.00 | 68 | 499.0 | 0.098 | 10550.0 | 16450 | 5.41 |
|  |  | S1(0～10) | Dark gray light loam | 4.42 | 14.8 | 4.59 | 70 | 163.5 | 0.087 | 191.5 | 367 | 10.30 |
|  |  | S2(10～30) | Brown light loam | 4.52 | 9.7 | 1.23 | 64 | 164.0 | 0.081 | 78.1 | 254 | 10.35 |
|  |  | S3(30～50) | Brown light loam | 4.68 | 5.9 | 1.94 | 73 | 156.5 | 0.052 | 46.3 | 270 | 10.15 |
|  |  | S4(50～70) | Brown light loam | 4.65 | 2.7 | 0.75 | 59 | 147.0 | 0.055 | 12.8 | 212 | 10.45 |
|  |  | S5(70～90) | Light brown sandy loam | 4.67 | 3.4 | 0.75 | 66 | 156.5 | 0.090 | 15.4 | 185 | 10.50 |
|  |  | S6(90～110) | Light brown sandy loam | 4.69 | 5.0 | 0.99 | 67 | 145.5 | 0.081 | 44.5 | 199 | 9.97 |
|  |  | S7(110～130) | Light brown sandy loam | 4.81 | 1.1 | 2.16 | 61 | 159.0 | 0.035 | 10.2 | 342 | 10.45 |
|  |  | S8(130～150) | Light brown sandy soil | 5.08 | 1.6 | 2.89 | 71 | 134.0 | 0.032 | 17.2 | 250 | 9.89 |
|  |  | S9(150～170) | Light brown sandy soil | 5.22 | 0.5 | 1.20 | 66 | 133.5 | 0.025 | 7.7 | 153 | 9.34 |
|  |  | S10(170～190) | Light brown sandy soil | 5.22 | 1.4 | 0.61 | 75 | 148.5 | 0.030 | 17.9 | 186 | 10.10 |
|  |  | S11(190～210) | Dark gray sandy soil containing black granular coal | 5.02 | 2.8 | 2.54 | 77 | 102.0 | 0.075 | 18.4 | 127 | 6.88 |
|  |  | Minimum value (except for the slag) | | 4.42 | 0.5 | 0.61 | 59 | 102.0 | 0.025 | 7.7 | 127 | 6.88 |
|  |  | Maximum value (except for the slag) | | 5.22 | 14.8 | 4.59 | 77 | 164.0 | 0.090 | 191.5 | 367 | 10.50 |
|  |  | Mean value (except for the slag) | | 4.82 | 4.45 | 1.79 | 68.09 | 146.36 | 0.058 | 41.82 | 231.36 | 9.85 |
| Slag-absent | In clastic rock region, covered with natural vegetation and without the slag, 100 meters away from the slag-covered profile | S1(0～10) | Brown light loam | 4.46 | 6.6 | 1.82 | 78 | 132.0 | 0.110 | 19.6 | 202 | 9.70 |
|  |  | S2(10～30) | Brown light loam | 4.56 | 6.7 | 0.64 | 72 | 127.5 | 0.105 | 14.7 | 149 | 9.78 |
|  |  | S3(30～55) | Brown light loam | 4.84 | 6.9 | 0.19 | 73 | 133.0 | 0.106 | 14.9 | 143 | 10.15 |
|  |  | S4(55～80) | Brown light loam | 4.98 | 7.8 | 0.17 | 94 | 135.0 | 0.069 | 16.2 | 144 | 10.35 |
|  |  | S5(80～105) | Light brown sandy loam | 5.09 | 7.7 | 0.15 | 76 | 134.0 | 0.058 | 15.6 | 142 | 10.20 |
|  |  | S6(105～130) | Light brown sandy loam | 5.06 | 5.5 | 0.14 | 76 | 129.0 | 0.068 | 15.2 | 139 | 10.20 |
|  |  | S7(130～155) | Light brown sandy soil | 5.12 | 3.7 | 0.17 | 76 | 130.5 | 0.044 | 8.4 | 129 | 9.77 |
|  |  | S8(155～180) | Light brown sandy soil | 5.09 | 1.8 | 0.20 | 72 | 130.0 | 0.027 | 5.7 | 135 | 8.93 |
|  |  | S9(180～205) | Light brown sandy soil | 5.07 | 2.6 | 0.21 | 68 | 118.0 | 0.051 | 9.1 | 133 | 8.68 |
|  |  | S10(205～225) | Dark gray sandy soil containing black granular coal | 5.09 | 0.2 | 0.83 | 49 | 84.5 | 0.046 | 10.3 | 61 | 5.10 |
|  |  | Minimum value | | 4.46 | 0.2 | 0.14 | 49 | 84.5 | 0.027 | 5.7 | 61 | 5.10 |
|  |  | Maximum value | | 5.12 | 7.8 | 1.82 | 94 | 135.0 | 0.110 | 19.6 | 202 | 10.35 |
|  |  | Mean value | | 4.94 | 4.95 | 0.45 | 73.40 | 125.35 | 0.068 | 12.97 | 137.70 | 9.29 |
| CCA | | | |  | 1.8 | 0.2 | 100 | 55 | 0.08 | 12.5 | 70 | 8.23 |
| SQV (pH<6.5) | | | |  | 40 | 0.3 | 150 | 50 | 0.3 | 250 | 200 |  |

Note: CCA means the average crustal content reference to Taylor (Taylor, 1964), SQV represents the risk-based screening values for the soil contamination of agricultural land (MEE and SAMR, 2018).

**Reference**

Taylor, S. R., 1964. Abundance of chemical elements in the continental crust: a new table. Geochimica Et Cosmochimica Acta, **28**(8), 1273-1285.

Ministry of Ecology and Environment of the People's Republic of China (MEE), State Administration for Market Regulation of the People's Republic of China (SAMR), 2018. Soil environmental quality risk control standard for soil contamination of agricultural land. GB 15168－2018, China Environment Press. (In Chinese)
